# REporting recommendations for tumour MARKer prognostic studies (REMARK)

**DOI:** 10.1038/sj.bjc.6602730

**Published:** 2005-08-16

**Authors:** A L Harris

**Affiliations:** 1 Cancer Research UK, Molecular Oncology Laboratories, Weatherall Institute of Molecular Medicine, John Radcliffe Hospital, Headington, Oxford OX3 9DS, UK; 2 Medical Oncology Unit, Churchill Hospital, Headington, Oxford OX3 7LJ, UK

One of the key ways to translate new basic discoveries into clinical relevance is the analysis of gene expression in tumour samples, relating this to various outcomes. These may include relapse-free survival and overall survival, sites of metastasis and, of course, differential expression in tumour *vs* normal is important to describe. The above are prognostic factors, but use of markers to predict which patients will benefit from increasingly complex and expensive therapies is potentially of high utility and a logical follow on, once patients with poor prognosis are selected. To achieve the goal of individualised medicine, with the selection of correct therapy for the molecular pathways in the tumour, this approach is needed.

When a new marker or protein is discovered and investigated in this way, there are often many failings in the initial phase of the development and validation. Examples include initial small studies that are made because the investigators do not have good access to clinical material. There may be many small studies, none of which have the individual statistical power to really test the significance of the marker. Owing to the small studies they may have wide variability in the statistical reliability and even opposite results. Very few of them seem to describe the number of cases that are needed to adequately test significantly the factor being examined.

The assays themselves, being research based, may be poorly validated and not well described, certainly not including the reproducibility interassay variation and other measures of reproducibility and standardisation. This is particularly the case in immunohistochemical studies but often with other quantitative assays such as Western blotting and ELISAs. These sources of variability may explain early controversies in the literature when new markers are described by different groups.

The paper by McShane *et al* (2005) published on pages 387–391 in this issue of BJC, represents several years of discussion from experts in this area, representing European and USA groups, who have produced a consensus on how to design and present these studies. These recommendations will be published simultaneously by several journals and produce a recommended standard that will help solve some of these problems.

Although their recommendations apply to prognostic factors, they are also applicable to predictive factors. Many important randomised trials now have 5–10 years follow-up, from which the basis for the current and future therapy is derived, for example, the adjuvant therapy of breast cancer. Defining the groups of patients who benefit from chemotherapy or endocrine therapy has substantial implications for patient selection, design of future trials and cost effectiveness. These trials have 1000s of patients in them and are an ideal way to test both predictive and prognostic factors. The only way predictive factors can really be tested is in randomised trials where one treatment is compared to another or no treatment and the value of a test to predict response to therapy would also need to be validated in further studies.

The development of well-characterised tissue microarrays has expedited the ability to analyse markers and pathways and many new techniques being applied to these, including extraction of DNA for comparative genome hybridisation and new methods to extract RNA, thus being able to carry out reverse transcription and quantitative polymerase chain reaction (rtPCR) assays to derive gene profiles (Paik *et al*, 2004). The latter study demonstrates how a rigorous approach in development of assay methodology can be applied to retrospective material effectively. Even larger numbers of samples can be studied with new techniques, providing not only greater statistical strength but also further technical challenges (Rimm, 2005).

It is particularly important that these types of studies are comparable and can be validated between studies to maximise information that will be available from them. However, these observations apply not just to these large retrospective analyses but also to new prospective studies and the validation of new markers on frozen material. Many basic techniques that are presented, including rtPCR, Western blotting, immunochemistry, really do not have a rigorous standardisation that will allow reproducibility.

To some extent this is a problem also generated by Journals that want to minimise the amount of space used in Methods Sections and therefore will prevent authors describing results in a way that would allow rigorous replication. However, one would hope that with the increasing access to web material, these detailed protocols would be available and indeed that this would be a requirement in the future for new markers, to be able to link the detailed validation to the short Methods Section in the papers.

Considering the increasing ethical issues and difficulties with accessing human material from trials prospectively and retrospectively (although these are being overcome), it is particularly important that when they are subjected to assay that the latter are of better quality, validated and reproducible.

It is interesting to note that where assays have major therapeutic implications then rapid, often commercial reliable testing does become available, for example use of Herceptin and FISH analysis of tumour samples. However, in spite of the use of endocrine therapy for over 30 years, the immunohistochemical analyses for oestrogen and progesterone receptors still do not seem to have an adequate international standardisation.

The BJC considers the publication of prognostic studies and predictive studies as an important component of translational research and we would expect authors submitting papers to this area to be aware of and to follow these guidelines. Further detailed evaluation of methodology and validation of assays can be accepted as supplementary material.
